# Modifiable Lifestyle Determinants of Hypertension and Cardiovascular Risk in Southeastern Nigeria: A Narrative Review From a Socioecological Perspective

**DOI:** 10.7759/cureus.109627

**Published:** 2026-05-25

**Authors:** CFC Ogbuefi, OL Ezika, KE Ogbuefi, NN Frank-Okeke

**Affiliations:** 1 Department of Family Medicine, Federal University Teaching Hospital, Owerri, NGA; 2 Department of Family Medicine, Federal Medical Centre, Umuahia, NGA

**Keywords:** cardiovascular risk, dietary sodium intake, hypertension, lifestyle modification, physical inactivity

## Abstract

Hypertension remains one of the leading contributors to cardiovascular disease and premature mortality in Nigeria and other low- and middle-income countries. Rapid urbanization, unhealthy dietary patterns, physical inactivity, and increasing sedentary behavior continue to drive rising cardiovascular risk across many communities. This narrative review examines the influence of modifiable lifestyle determinants of hypertension and cardiovascular disease within Southeastern Nigeria from a socioecological perspective. Relevant literature relating to physical inactivity, sedentary behavior, dietary sodium intake, health literacy, socioeconomic influences, and preventive healthcare systems was reviewed and synthesized using thematic analysis. The evidence demonstrates that cardiovascular risk is shaped not only by individual behaviors but also by broader interpersonal, environmental, and health-system factors that influence prevention and long-term disease control. The review highlights the importance of context-specific public health interventions that integrate lifestyle modification, community education, improved primary healthcare delivery, and strengthened prevention policies. Addressing hypertension in resource-limited settings will require coordinated multilevel strategies capable of supporting sustainable cardiovascular risk reduction.

## Introduction and background

Cardiovascular disease remains the leading cause of mortality worldwide, accounting for an estimated 17.9 million deaths annually and approximately one-third of all global deaths [1]. Among the major contributors to this burden, hypertension is widely recognized as the most important modifiable risk factor because of its strong association with ischemic heart disease, stroke, heart failure, chronic kidney disease, and premature mortality [2]. Although effective pharmacological and non-pharmacological interventions are available, hypertension prevention, diagnosis, treatment, and control remain inadequate in many settings, particularly in low- and middle-income countries where structural, economic, and health-system barriers continue to limit implementation of evidence-based care [1].

Over the past several decades, the global epidemiology of hypertension has shifted substantially. While many high-income countries have achieved gradual improvements in awareness, treatment, and blood pressure control through population-level prevention strategies and stronger health systems, low- and middle-income countries now bear a disproportionate share of the burden [2]. This shift has been driven by rapid urbanization, demographic transition, population aging, reduced physical activity, changing occupational patterns, increased sedentary behavior, and growing consumption of processed and sodium-rich foods. As a result, hypertension has evolved from a predominantly clinical concern into a major public health challenge with wide-reaching social and economic consequences.

Nigeria exemplifies this transition. As the most populous country in Africa, Nigeria is experiencing a rising burden of non-communicable diseases, with hypertension contributing substantially to cardiovascular morbidity and mortality. A systematic analysis of evidence from 1995 to 2020 reported that more than one-third of Nigerian adults are hypertensive, while awareness, treatment, and control remain persistently suboptimal [3]. This pattern reflects not only the growing prevalence of elevated blood pressure but also major deficiencies in preventive healthcare delivery, early detection, continuity of care, and long-term risk reduction.

Evidence from southeastern Nigeria, including Enugu State, mirrors these national trends while also revealing important regional concerns. Community-based studies have reported a high prevalence of cardiovascular risk factors and substantial levels of undiagnosed hypertension among adults in southeastern Nigeria [4,5]. Facility-based evidence from South-East Nigeria similarly shows that many adults presenting for unrelated medical conditions have previously unrecognized hypertension, highlighting missed opportunities for routine screening and preventive intervention within healthcare settings [6]. Particularly concerning is the emerging evidence that hypertension and prehypertension are increasingly being documented among younger adults in Enugu State, suggesting earlier and more prolonged exposure to cardiovascular risk [7,8].

Lifestyle-related factors play a central role in the development and progression of hypertension within the Nigerian context. Physical inactivity has consistently been associated with elevated blood pressure and adverse cardiovascular outcomes, and low adherence to recommended physical activity remains common in both urban and occupational populations [9-13]. Sedentary behavior, which is conceptually distinct from physical inactivity, has also emerged as an independent cardiovascular risk factor, with prolonged sitting linked to impaired metabolic regulation and increased cardiovascular risk even among individuals who report intermittent exercise [2]. In many Nigerian settings, these patterns are reinforced by desk-based occupations, prolonged screen exposure, motorized transportation, and limited access to safe or affordable spaces for recreation and exercise [9,12].

Dietary behavior is another major determinant of hypertension risk. High sodium intake remains widespread in many Nigerian communities because of traditional cooking practices, frequent use of seasoning products, consumption of processed foods, and limited awareness of recommended salt intake thresholds [10,11]. Qualitative evidence from southern Nigeria has shown that barriers to salt reduction include habitual taste preferences, low risk perception, and practical difficulties in changing long-established household food practices. These challenges are further shaped by cultural expectations around food preparation and acceptability, which may reduce the effectiveness of generic dietary advice if the local context is not adequately considered. Although national sodium-reduction strategies have been developed, implementation remains inconsistent because of weak policy enforcement, limited public education, and variable translation of policy into routine practice [14,15].

Importantly, the burden of hypertension cannot be explained by behavior alone. Socioeconomic disadvantage, out-of-pocket healthcare costs, low health literacy, and fragmented preventive services all contribute to delayed diagnosis, poor treatment adherence, and inadequate long-term blood pressure control. Effective prevention therefore requires a broader public health approach that integrates risk-factor modification with strengthened primary care, improved health communication, and better implementation of national and global prevention frameworks [16]. The World Health Organization HEARTS technical package, for example, emphasizes standardized cardiovascular risk management, community engagement, and delivery of preventive services through primary healthcare systems, making it especially relevant in resource-limited settings [17].

The socioecological model offers a useful framework for understanding these interacting influences. Rather than viewing hypertension solely as the result of individual lifestyle choices, the model recognizes that behavior is shaped by interpersonal relationships, community environments, institutional structures, and policy conditions. In settings such as southeastern Nigeria, this perspective is particularly valuable because it helps explain why risk behaviors persist even when basic awareness exists and why sustainable prevention requires coordinated action at multiple levels.

This narrative review, therefore, examines the influence of modifiable lifestyle determinants, particularly physical inactivity, sedentary behavior, and excessive dietary sodium intake, on hypertension and cardiovascular risk among adults in Southeastern Nigeria [9-12,14,15]. Using the socioecological model as an organizing framework, the review synthesizes evidence across individual, interpersonal, community, and policy levels in order to identify practical and context-relevant strategies for cardiovascular risk reduction in a resource-limited setting. This article is a narrative review using thematic synthesis to organize evidence on modifiable lifestyle determinants of hypertension in Southeastern Nigeria. In doing so, it aims to provide evidence-based insights that can inform public health intervention design, primary healthcare strengthening, and local policy development (Table 1).

**Table 1:** Key modifiable cardiovascular risk factors addressed in this review This table summarizes the principal modifiable lifestyle-related cardiovascular risk factors discussed in this review and highlights their corresponding prevention focus areas within the context of hypertension reduction and cardiovascular disease prevention.

Risk Factor	Description	Prevention Focus
Physical inactivity	Low levels of moderate-to-vigorous activity	Community exercise promotion
Sedentary behavior	Prolonged sitting and screen time	Behavioral modification
High salt intake	Excess sodium from cooking and processed foods	Dietary education
Low health literacy	Limited understanding of hypertension prevention	Risk communication

## Review

Methods

This study was conducted as a narrative review using thematic synthesis rather than a systematic review or meta-analysis. The review examined the relationship between modifiable lifestyle determinants and hypertension in Nigeria, with particular attention to Southeastern Nigeria. The review focused on recent evidence relating to hypertension epidemiology, physical inactivity, sedentary behavior, dietary sodium exposure, socioeconomic influences, health literacy, and health-system factors relevant to cardiovascular disease prevention in resource-limited settings.

Search Strategy

Literature was searched in PubMed, Google Scholar, and selected institutional repositories for publications from 2019 to 2026 using combinations of the terms: hypertension, cardiovascular disease, physical inactivity, sedentary behavior, sodium intake, lifestyle modification, Nigeria, Enugu State, southeastern Nigeria, and sub-Saharan Africa.

Study Selection

The search identified approximately 145 records. After duplicate removal and title/abstract screening, 52 records were assessed for full-text relevance, and 32 studies were included in the final narrative synthesis.

Thematic Synthesis

Included studies were read iteratively and grouped according to recurring concepts. Themes were developed from patterns in the literature and organized deductively using the socioecological model across individual, interpersonal, community, and policy levels. Initial coding and theme grouping were reviewed by the authors, and disagreements were resolved through discussion.

A literature search was conducted using PubMed, Google Scholar, and selected institutional repositories. The search was limited to literature published between 2019 and 2026 in order to capture recent epidemiological trends and contemporary prevention strategies. Search terms included hypertension, cardiovascular disease, physical inactivity, sedentary behavior, prolonged sitting, dietary salt intake, sodium consumption, lifestyle modification, Nigeria, Enugu State, Southeastern Nigeria, and sub-Saharan Africa.

The literature search initially identified approximately 145 records across all databases and repositories. After removal of duplicates and screening for relevance to hypertension epidemiology, lifestyle determinants, cardiovascular risk, and prevention strategies in Nigeria and comparable low- and middle-income settings, 52 sources were considered potentially eligible for full-text review. Following full-text assessment, 32 references were ultimately included in the final narrative synthesis based on their relevance to the objectives of the review and their contribution to the thematic analysis. Although this review did not follow a formal systematic review protocol or PRISMA framework, efforts were made to ensure balanced representation of epidemiological evidence, behavioral risk factors, health-system influences, and public health intervention strategies relevant to resource-limited settings.

Sources were considered eligible if they addressed at least one of the following domains: hypertension prevalence, prehypertension, modifiable lifestyle-related cardiovascular risk factors, sodium-reduction practices, preventive interventions, health literacy, healthcare access, or policy and health-system determinants. Priority was given to peer-reviewed original research, systematic reviews, narrative reviews, policy documents, and relevant public health reports addressing Nigeria or comparable low- and middle-income settings. Studies were excluded if they were clearly unrelated to the objectives of the review, were conducted in settings with limited contextual relevance, or did not contribute meaningful insight into the lifestyle and structural determinants of hypertension.

**Figure 1:**
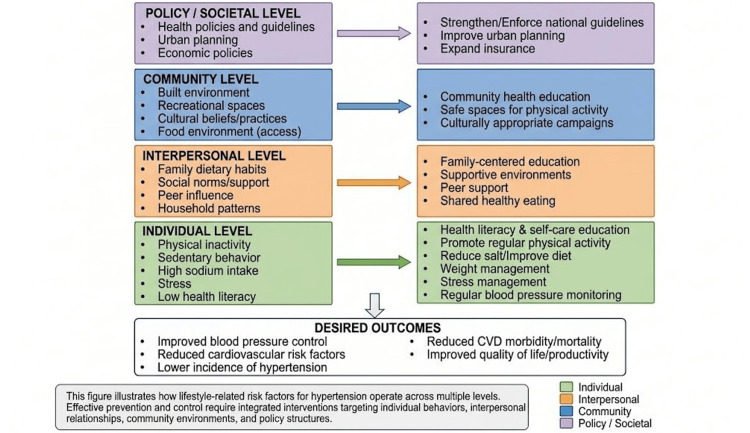
Socioecological model of lifestyle determinants of hypertension in Southeastern, Nigeria. This figure illustrates the multilevel determinants influencing hypertension risk, including individual, interpersonal, community, and policy-level factors, alongside corresponding intervention strategies for cardiovascular disease prevention. Image Credits: CFC Ogbuefi, OL Ezika, KE Ogbuefi, and NN Frank-Okeke. Image created manually by the authors using Python (Matplotlib; Python Software Foundation, Wilmington, DE, USA). No AI assistance was utilized in its creation. CVD: cardiovascular disease

The review was guided by the socioecological model, which was used as the analytical framework for organizing the evidence across individual, interpersonal, community, and policy levels (Figure 1). This approach was selected because it provides a practical structure for understanding how behavioral risk factors interact with social relationships, environmental conditions, and broader health-system and policy influences in shaping cardiovascular risk in resource-limited settings. Because this is a narrative review, no meta-analysis, meta-regression, or formal quantitative pooling was performed.

**Figure 2:**
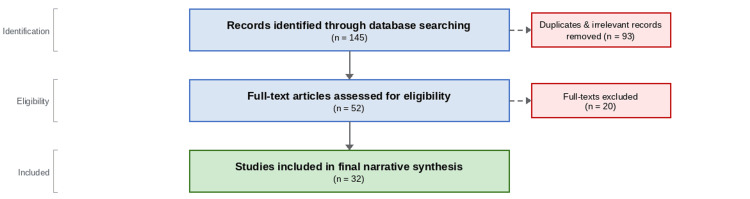
Literature search and study selection process. Flow diagram summarizing the identification, screening, eligibility assessment, and final inclusion of studies used in this narrative review. Image Credits: CFC Ogbuefi, OL Ezika, KE Ogbuefi, and NN Frank-Okeke. Image created manually by the authors using Python (Matplotlib; Python Software Foundation, Wilmington, DE, USA). No AI assistance was utilized in its creation. n: number

Figure 2 summarizes the literature search and study selection process used in this narrative review. Data from the selected literature were synthesized thematically rather than quantitatively. The analysis focused on four broad domains: the burden of hypertension, lifestyle and behavioral risk factors, socioeconomic and health-system influences, and implementation gaps in prevention and control strategies. Representative studies included in the narrative synthesis are summarized in Table 2. These studies were selected because they were considered representative of the major thematic domains of the review, including hypertension epidemiology, physical inactivity, dietary sodium exposure, implementation challenges, and cardiovascular prevention strategies relevant to Nigeria and comparable low-resource settings.

**Table 2:** Representative studies included in the narrative review: author, year, ref, study design, sample size, study population, setting, and key findings. This table summarizes selected studies included in the narrative synthesis, highlighting their study focus, setting, and principal findings relevant to hypertension and cardiovascular risk determinants in Nigeria and comparable settings.

Author, Year [Ref]	Study Design	Sample Size (n)	Study Population	Setting	Key Findings
Adeloye et al., 2021 [3]	Systematic review and random-effects meta-analysis	53 studies pooled (n = 78,949)	Nigerian adults	Nigeria (national pooled data)	Hypertension cases increased substantially between 1995 and 2020, while awareness, treatment, and blood pressure control rates remained low.
Amuzie et al., 2022 [12]	Cross-sectional study	n = 440	Civil servants selected via multistage sampling	Abia State, Southeastern Nigeria	Physical inactivity and sedentary behavior were strongly associated with environmental and structural cardiovascular risk factors.
Sanuade et al., (2023) [15]	Qualitative study (interviews and focus groups)	23 interviews and 5 focus group discussions	Regulators, food producers, consumers, retailers, academics, and healthcare workers	Nigeria (national implementation analysis)	Major implementation gaps, resource limitations, and monitoring barriers affected national sodium reduction strategies.
Nwoke et al., 2024 [7]	Cross-sectional study	n = 279	Medical students aged 18–35 years	Enugu State, Southeastern Nigeria	Prehypertension and hypertension were increasingly identified among young adults and were associated with elevated BMI and waist circumference.
Oku et al., 2024 [10]	Qualitative focus group discussion study	8 focus groups (n = 74)	Hypertensive adults with high dietary salt intake	Southern Nigeria (hospital-based)	Cultural food practices, habitual taste preferences, and poor risk perception limited dietary salt reduction.
Leeyio et al., 2025 [18]	Systematic review and meta-analysis	8 clinical trials pooled (n = 1,112)	Hypertensive adults receiving antihypertensive therapy	Africa (regional pooled analysis)	Structured aerobic exercise significantly reduced systolic and diastolic blood pressure among adults with hypertension.

Review findings

Burden of Hypertension

The reviewed literature consistently demonstrates that hypertension remains highly prevalent in Nigeria and continues to represent a major public health challenge [3]. National evidence indicates that more than one-third of Nigerian adults are affected, with substantial variation across regions, age groups, and patterns of healthcare access [3]. Despite the magnitude of this burden, awareness, treatment, and blood pressure control remain inadequate, with many individuals either unaware of their condition or not receiving sustained care sufficient to achieve optimal control [3].

Evidence from southeastern Nigeria reflects these broader national patterns [4-6]. Community-based studies have documented a high prevalence of hypertension and related cardiovascular risk factors among adult populations in the region [4,5]. Hospital-based studies from South-East Nigeria further show that a significant number of adults presenting for unrelated complaints have previously undiagnosed hypertension, underscoring persistent gaps in routine screening and early detection [6]. These findings suggest that many opportunities for prevention and timely intervention are still being missed at both community and facility levels [4-6].

Recent studies have raised additional concern about the shifting age distribution of hypertension [8]. Evidence from Enugu State has demonstrated increasing prevalence of prehypertension and hypertension among younger adults, suggesting earlier exposure to modifiable risk factors and the possibility of a longer lifetime burden of cardiovascular disease [7,8]. This trend is particularly important because the earlier onset of elevated blood pressure may increase cumulative vascular injury and future risk of stroke, heart failure, kidney disease, and other cardiovascular complications [2].

Lifestyle and Behavioral Risk Factors

Physical inactivity remains one of the most important modifiable contributors to hypertension and cardiovascular risk in Nigeria [9,12]. Low participation in moderate-to-vigorous physical activity has been reported in urban and occupational populations, where sedentary work patterns, environmental barriers, and limited access to recreational facilities often reduce opportunities for regular movement [9,12]. Evidence from African populations also shows that aerobic activity can significantly reduce blood pressure among adults with hypertension, reinforcing the preventive and therapeutic importance of regular physical activity [18].

Sedentary behavior is a related but distinct risk factor that warrants separate consideration [19]. Prolonged sitting has been associated with impaired metabolic regulation, reduced vascular function, and greater cardiovascular risk, even among individuals who engage in some physical activity [19]. In many Nigerian urban settings, sedentary behavior is increasingly reinforced by desk-based occupations, long commuting times, motorized transportation, prolonged screen exposure, and reduced walkability of the built environment [9,10]. These patterns suggest that interventions should address both inadequate exercise and excessive sitting time rather than treating them as interchangeable concepts.

Dietary exposure, particularly excess sodium intake, also plays a major role in hypertension development and progression [10-12]. High salt consumption in Nigerian settings is influenced by traditional cooking practices, frequent use of salt-rich seasoning products, processed food consumption, and low awareness of recommended sodium thresholds. Qualitative evidence from southern Nigeria indicates that salt reduction is often constrained by entrenched taste preferences, poor risk perception, and limited practical knowledge of healthier alternatives [20]. Cultural food practices may further reinforce high sodium consumption and reduce adherence to standard dietary advice when interventions are not adapted to the local context [11,12].

These behavioral risk factors rarely occur in isolation. Instead, physical inactivity, prolonged sitting, and unhealthy dietary habits often coexist and interact within the same social and environmental setting, thereby amplifying cardiovascular risk [9-12]. Taken together, the reviewed evidence supports the need for integrated preventive approaches that address multiple lifestyle determinants simultaneously rather than focusing on a single behavior in isolation.

Socioeconomic and Health-System Factors

Socioeconomic constraints significantly influence both hypertension prevention and long-term disease control in Nigeria. High out-of-pocket healthcare costs limit access to blood pressure screening, clinical review, medications, and follow-up care, thereby contributing to delayed diagnosis and poor treatment continuity [21]. Financial barriers may also reduce patients’ ability to maintain recommended diets, engage in structured preventive care, or consistently adhere to prescribed therapy [22].

Health literacy is another important determinant of cardiovascular outcomes. Limited understanding of hypertension, its long-term complications, and the role of lifestyle modification reduces the likelihood that individuals will participate in routine screening, adopt preventive behaviors, or remain adherent to treatment plans [23]. Poor health literacy may also weaken risk perception, especially in asymptomatic individuals, thereby encouraging late presentation after complications have already developed.

Health-system limitations further compound these challenges. Preventive services remain underutilized in many settings, and blood pressure measurement is still not consistently integrated into all healthcare encounters despite its low cost and public health value [3]. Workforce shortages, fragmented service delivery, inadequate counseling time, and weak continuity of care all limit effective hypertension prevention and management. Collectively, these findings indicate that poor cardiovascular outcomes in Nigeria are shaped not only by individual behavior but also by broader social, economic, and structural determinants that constrain the delivery and uptake of preventive care [3,16,21-23].

Implementation Gaps

Although national and international frameworks for hypertension prevention and cardiovascular risk reduction are available, implementation remains inconsistent in many Nigerian settings [15-17]. Stakeholder evidence suggests that sodium-reduction strategies, public health education efforts, and preventive service delivery are often weakened by insufficient resources, inconsistent policy translation, and limited monitoring of implementation outcomes. In practice, these gaps manifest as irregular screening, inadequate lifestyle counseling, weak public awareness, poor follow-up systems, and limited enforcement of preventive policies [15-17].

These implementation challenges are especially important in resource-limited settings such as southeastern Nigeria, where the success of hypertension prevention depends not only on the availability of recommendations but also on the capacity of communities and health systems to operationalize them. As a result, many evidence-based interventions remain underused or inconsistently applied, reducing their potential impact on population-level blood pressure control and cardiovascular disease prevention.

Discussion

The findings of this review underscore that hypertension in Nigeria is not simply an isolated clinical diagnosis but a multifactorial public health problem shaped by the interaction of behavioral, environmental, socioeconomic, and health-system determinants. Because this is a narrative review, the findings are synthesized qualitatively rather than pooled statistically. The high prevalence of hypertension, combined with low levels of awareness, treatment, and blood pressure control, highlights persistent gaps in prevention and chronic disease management [2,8]. In southeastern Nigeria and related settings, these gaps are further compounded by missed opportunities for early detection and by the growing burden of elevated blood pressure among younger adults [24]. Taken together, these patterns suggest that hypertension in Nigeria is increasingly driven by sustained exposure to modifiable risks within contexts that do not adequately support prevention or long-term control [5,7,10,11].

A central finding of the reviewed evidence is the prominent role of lifestyle-related risk factors, particularly physical inactivity, prolonged sedentary behavior, and high dietary sodium intake [9-12]. These behaviors are especially important because they often cluster within the same individuals and communities, thereby amplifying cumulative cardiovascular risk. In many urban and semi-urban Nigerian settings, reduced routine movement, occupational sitting, motorized transport, and sodium-rich dietary habits are becoming progressively normalized as part of daily life [12,13,20,22]. This reinforces the need to view lifestyle determinants not merely as personal choices but as patterns shaped by changing social environments and constrained health-promoting alternatives.

The emergence of hypertension among younger adults deserves particular attention. Recent studies from Enugu State and other Nigerian populations suggest that prehypertension and hypertension are increasingly present in early adult life, including among educated and apparently healthy groups such as students [24]. This trend carries important long-term implications because an earlier onset of elevated blood pressure may lead to a longer cumulative exposure to vascular injury and a greater lifetime risk of stroke, heart failure, chronic kidney disease, and other cardiovascular complications [2]. Community-based evidence from Enugu further indicates coexistence of hypertension with other cardiometabolic disorders such as diabetes mellitus, suggesting that the burden of risk is often compounded rather than isolated [25].

The reviewed evidence also supports a broader biological rationale for aggressive lifestyle prevention. Beyond their direct association with elevated blood pressure, unhealthy behavioral patterns contribute to adverse metabolic and vascular changes that promote cardiovascular disease progression [25]. Chronic low-grade inflammation has increasingly been recognized as one of the mechanisms linking unhealthy lifestyles to cardiovascular pathology, providing further support for preventive strategies that target diet, activity, and other modifiable exposures early in the disease pathway [26]. In this sense, effective prevention of hypertension is also likely to reduce downstream cardiometabolic and inflammatory risk.

An important implication of this review is that the continuing burden of hypertension in Nigeria is not explained only by insufficient awareness at the individual level but also by weak implementation of already established prevention strategies [15-17]. Although national guidelines, WHO recommendations, and structured cardiovascular prevention frameworks exist, their routine application remains inconsistent across many health settings [15-17]. In practice, this inconsistency is reflected in limited opportunistic screening, inadequate counseling on physical activity and diet, poor follow-up systems, weak public awareness campaigns, and suboptimal policy enforcement related to sodium reduction and non-communicable disease prevention. The result is a persistent gap between what is known to reduce cardiovascular risk and what is actually delivered in everyday practice.

The socioecological model provides a useful lens for understanding why this gap persists. This model was used deductively to organize evidence across individual, interpersonal, community, and policy levels, while themes were refined iteratively during reading and comparison of studies. This means that hypertension prevention cannot rely exclusively on advising individuals to exercise more or consume less salt. Rather, meaningful change requires multilevel interventions that modify the contexts in which these behaviors occur, including household habits, community infrastructure, primary healthcare delivery, and public policy.

Based on the reviewed evidence, this study proposes a context-adapted multilevel prevention framework for hypertension reduction in southeastern Nigeria. At the individual level, interventions should prioritize health literacy improvement, dietary sodium reduction, and promotion of routine physical activity. At the interpersonal level, family-based dietary modification and peer-supported lifestyle counseling may improve long-term adherence to preventive behaviors. Community-level strategies should focus on improving opportunities for physical activity through safer walkable environments, workplace movement initiatives, and culturally appropriate cardiovascular health education campaigns. At the policy and health-system level, strengthening routine blood pressure screening, improving continuity within primary healthcare systems, expanding affordability of preventive care, and enhancing implementation of national sodium-reduction strategies are likely to produce broader population-level cardiovascular benefits. This multilevel framework highlights the importance of integrating behavioral, environmental, and structural interventions rather than relying exclusively on individual-level counseling approaches [15-17,21-23,27]. 

Public health responses should therefore prioritize interventions that are both evidence-based and context-sensitive. Community-based education remains essential for improving understanding of hypertension, its risk factors, and the importance of lifestyle modification. Such education is more likely to be effective when delivered in culturally appropriate formats, local languages, and practical messages that translate risk information into feasible daily actions [27]. Structured community-based and primary care educational models may be particularly valuable in settings where physician time is limited, and continuity of counseling is needed [27].

Promotion of physical activity should similarly move beyond generic recommendations. Counseling during clinic encounters is important, but it is unlikely to be sufficient in the absence of supportive environments. Creating safer and more accessible opportunities for walking, reducing prolonged sitting in workplaces and educational settings, and encouraging routine rather than purely recreational movement are practical approaches that may be more achievable in resource-limited settings [18,19]. Evidence from Nigerian medical student populations also suggests that even among future health professionals, the gap between knowledge and preventive practice remains important, further emphasizing the need for behavior-supportive environments rather than information alone [28].

Dietary sodium reduction represents another area where multilevel intervention is required. Individual counseling about salt restriction is important, but its effectiveness will remain limited if broader household, cultural, and food-system influences are not addressed. Public education should therefore be combined with stronger implementation of sodium-reduction policies, clearer labeling and messaging, and locally adapted strategies that acknowledge the culinary and social significance of food practices [15,16]. Without such contextualization, dietary advice may remain technically correct but behaviorally ineffective [20].

Improved access to affordable and continuous healthcare is also critical. High out-of-pocket spending remains a major barrier to hypertension detection, treatment, and follow-up in Nigeria. Financial strain may reduce clinic attendance, medication adherence, and the ability to sustain recommended behavioral changes over time [21]. Expanding routine blood pressure measurement across healthcare encounters, strengthening continuity within primary care, and improving access for high-risk individuals, including those with a family history of cardiovascular disease, may help improve earlier diagnosis and more sustained prevention [29].

Emerging digital approaches may offer additional support for cardiovascular prevention, although they should be considered complementary rather than central at present. Mobile health interventions have shown potential for promoting physical activity, reducing sedentary behavior, and improving patient engagement through reminders and self-monitoring tools [30]. In addition, artificial intelligence-driven risk assessment frameworks have been proposed as future tools for earlier and more individualized cardiovascular risk detection, although such models still require validation, contextual adaptation, and feasibility testing before wider application in routine care [31]. Wearable diagnostic technologies may also expand future opportunities for early cardiovascular surveillance, but their role in low-resource settings will depend on cost, accessibility, and health-system integration [32].

This review has several limitations. As a narrative review, it is subject to selection bias and does not provide quantitative pooled estimates. The search and synthesis process was structured but not registered as a systematic review protocol, and no formal risk-of-bias tool was applied to individual studies. In addition, evidence specific to Southeastern Nigeria remains limited, which may affect generalizability. Accordingly, the findings should be interpreted as a context-informed thematic synthesis rather than an exhaustive systematic appraisal of the literature.

Future research should focus on generating more local epidemiologic evidence from Southeast Nigeria and similar settings while also evaluating community-based and primary care-based interventions that address physical inactivity, sedentary behavior, dietary sodium exposure, and barriers to sustained blood pressure control. More implementation-focused research is also needed to determine how existing guidelines can be adapted into feasible and scalable strategies within routine Nigerian healthcare and community systems.

## Conclusions

Hypertension remains a major and preventable contributor to cardiovascular disease in Nigeria, with modifiable lifestyle factors such as physical inactivity, sedentary behavior, and excessive dietary sodium intake playing important roles in cardiovascular risk development. This review demonstrates that these behaviors are influenced not only by individual choices but also by broader social, environmental, and health-system conditions that affect prevention and long-term disease control.

Sustainable reduction in cardiovascular risk within southeastern Nigeria will require coordinated public health strategies that combine lifestyle education, community-based prevention, improved primary healthcare delivery, and stronger implementation of cardiovascular prevention policies. These recommendations are based on qualitative thematic synthesis rather than quantitative meta-analysis. Greater emphasis on preventive care and context-sensitive interventions may help reduce the growing burden of hypertension in resource-limited settings.
